# Introduction of a prognostic biomarker to strengthen risk stratification of acutely admitted patients: rationale and design of the TRIAGE III cluster randomized interventional trial

**DOI:** 10.1186/s13049-016-0290-8

**Published:** 2016-08-05

**Authors:** Andreas Sandø, Martin Schultz, Jesper Eugen-Olsen, Lars Simon Rasmussen, Lars Køber, Erik Kjøller, Birgitte Nybo Jensen, Lisbet Ravn, Theis Lange, Kasper Iversen

**Affiliations:** 1 Department of Cardiology, Herlev Hospital, University of Copenhagen, Herlev Ringvej 75, 2730 Herlev, Denmark; 2 Clinical Research Centre, Hvidovre Hospital, University of Copenhagen, Kettegård Alle 30, 2650 Hvidovre, Denmark; 3 Department of Anaesthesia, Rigshospitalet, University of Copenhagen, Blegdamsvej 9, 2100 Copenhagen, Denmark; 4 Department of Cardiology, Rigshospitalet, University of Copenhagen, Blegdamsvej 9, 2100 Copenhagen, Denmark; 5 Department of Emergency Medicine, Bispebjerg Hospital, University of Copenhagen, Bispebjerg Bakke 23, 2400 Copenhagen, Denmark; 6 Department of Emergency Medicine, Herlev Hospital, University of Copenhagen, Herlev Ringvej 75, 2730 Herlev, Denmark; 7 Department of Public Health, University of Copenhagen, Øster Farimagsgade 5, 1014 Copenhagen, Denmark

**Keywords:** Risk stratification, Triage, Biomarkers, SuPAR, Emergency medicine, Acute patients

## Abstract

**Background:**

Several biomarkers have shown to carry prognostic value beyond current triage algorithms and may aid in initial risk stratification of patients in the emergency department (ED). It has yet to be established if information provided by biomarkers can be used to prevent serious complications or deaths. Our aim is to determine whether measurement of the blood level of the biomarker *soluble urokinase plasminogen activator receptor* (suPAR) can enhance early risk stratification leading to reduced mortality, lower rate of complications, and improved patient flow in acutely admitted adult patients at the ED. The main hypothesis is that the availability of suPAR can reduce all-cause mortality, assessed at least 10 months after admission, by drawing attention towards patients with an unrecognized high risk, leading to improved diagnostics and treatment.

**Methods:**

The study is designed as a cross-over cluster randomized interventional trial. SuPAR is measured within 2 h after admission and immediately reported to the treating physicians in the ED. All ED physicians are educated in the prognostic capabilities of suPAR prior to the inclusion period. The inclusion period began January 11^th^ 2016 and ends June 6^th^ 2016. The study aims to include 10.000 patients in both the interventional and control arm. The results will be presented in 2017.

**Discussion:**

The present article aims to describe the design and rationale of the TRIAGE III study that will investigate whether the availability of prognostic information can improve outcome in acutely admitted patients. This might have an impact on health care organization and decision-making.

**Trial registration:**

The trial is registered at clinicaltrials.gov (ID NCT02643459, November 13, 2015) and at the Danish Data Protection agency (ID HGH-2015-042 I-Suite no. 04087).

## Background

Risk stratification with triage of patients admitted to the emergency department (ED) plays a pivotal role in ensuring that the most acutely sick patients are cared for first [1]. Existing triage algorithms are all based on a combination of the patients’ vital signs and primary symptoms [1, 2]. Several retrospective studies have identified biomarkers that provide prognostic information which goes beyond the current triage utilized in the EDs [3–10].

Whether the implementation of a prognostic biomarker in initial risk stratification of acutely admitted patients translates into better management and treatment of high risk patients and *actually* decreases mortality, morbidity, admissions or readmissions has yet to be shown.

*Soluble urokinase plasminogen activator receptor* (suPAR) is a prognostic biomarker with potential use in the EDs. The suPAR blood level reflects immune activation and it is strongly associated with mortality and presence, prognosis and severity of a broad variety of acute and chronic diseases [8, 11–18], and it is also a predictor of disease development in the general population ([19]; Rasmussen et al.: suPAR in Acute Care: Associated with Disease Severity, Readmission, and Mortality, in review). As an unspecific biomarker with strong prognostic value across morbidities, suPAR might be a useful biomarker for risk stratification in an ED, as the staff can target intervention, resources, and clinical focus where most beneficial.

The primary aim of the study is to evaluate whether the availability of an unspecific biomarker (suPAR) as a supplement to risk stratification of unselected acutely admitted patients can reduce all-cause mortality.

## Design

### Study design

The TRIAGE III study is an open cross-over cluster randomized, parallel interventional two center trial on the effect of suPAR level measurements in the ED. Enrolment began January 11th 2016 and inclusion is planned to be completed June 6th 2016. The study is designed as two clusters (two EDs at two hospitals in the capital region of Denmark) around three cycles of three weeks in a 1:1 allocation ratio of intervention versus control at each hospital (Table 1). SuPAR level measurement is included in the standard blood work of all eligible patients acutely admitted in the interventional period. In the control period the suPAR level is not measured.

**Table 1:** Trial structure

Cycle	1	2	3	4	5	6
Cluster 1	+suPAR	Control	+suPAR	Control	+suPAR	Control
Cluster 2	Control	+suPAR	Control	+suPAR	Control	+suPAR

Each cycle consists of three weeks with or without suPAR measurements in the ED

*ED* emergency department, *SuPAR* soluble urokinase plasminogen activator receptor

#### Study hypothesis

The main hypothesis is that the introduction, fast measurement and immediate reporting of the suPAR level to attending physicians in the EDs will be associated with a reduction in all-cause mortality at least 10 months after admission.

#### Outcomes

The primary outcome is all-cause mortality assessed on 6^th^ of April 2017, 10 months after inclusion of the last patient. Secondary outcomes are listed in Table 2.

**Table 2:** Secondary outcomes

Secondary outcomes	Timeframe
All-cause mortality after index admission	30 days
Proportion of patients discharged from the ED	24 h
Proportion of patients admitted to the ICU	30 days
Incidence of new cancer diagnoses	10 months after inclusion ends
Admission length	
Readmissions rate	30 days

A readmission is defined as any subsequent patient hospital admission within 30 days of index admission

*ED* emergency department, *ICU* intensive care unit

#### Eligibility criteria

All patients presenting acutely to the ED and have blood work done including hemoglobin, C-reactive protein and creatinine within 6 h of registration are included. Patients presenting in Pediatric, Gynecological or Obstetric units are excluded.

#### Quantification of suPAR

Blood samples (6 mL EDTA plasma tubes) for measurement of plasma suPAR are drawn along with the routine blood work. For quantification of suPAR, blood collection tubes are spun for 60 s at 6000 RPM. 10 μL of plasma is added to a prefabricated tube containing 100 μL of running buffer. Using a 60 μL pipette, the plasma and buffer are mixed by pipetting the solution up and down 5 times. From this mixture, 60 μL is added to the suPARnostic® Quick Triage stick, a lateral flow device. After 20 min, the lateral flow device is visually inspected for test and control line, and the suPAR test line quantified using a suPARnostic Quick test devise reader (Qiagen, Germany) [20].

According to the test manufacturer (ViroGates A/S, Birkeroed, Denmark), the limit of Detection (LOD) for the suPARnostic quick test is 0.3 ng/mL. The limit of quantification (LOQ) is 2 ng/mL defined at the lowest concentration with a CV% that does not exceed 25 %. The intra- and interserial measured CV% on 5 samples x 4 concentrations (2.0; 4.0; 8.4; 13.7 ng/mL) measured on the same day or with 5 days interval is less than 25 %. The r^2^ of the suPARnostic Quick Test compared to the suPARnostic ELISA is 0.875 [21]. Analysis of suPAR level is handled by trained medical students according to the manufacturer’s instructions, available on-site full-time for non-stop inclusion of eligible patients. All suPAR levels are analyzed as quickly as possible and always within two hours following blood sampling and immediately reported.

#### Information to physicians

The suPAR level is presented to the attending physicians through the electronical systems LABKA, OPUS and Cetrea. LABKA II (v. 2.5.0.H2, Computer Sciences Corporation (CSC)) is the clinical laboratory information system used to request blood work and view results from laboratory analysis. OPUS (OPUS Arbejdsplads, v. 2.5.0.0, Computer Sciences Corporation (CSC) is the electronical database of medical records. The emergency wards in the EDs are monitored by the Cetrea system presented by several large screen monitors in the ED and presents a rough overview of the ward (patient data and status, possible diagnosis, route of admission) used by physicians and nurses.

**Fig. 1:**
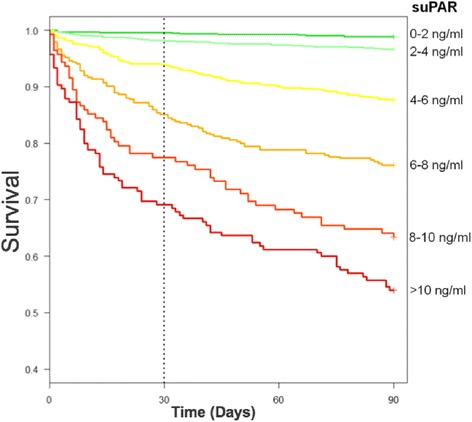
Kaplan-Meier plot of survival stratified by age- and sex-specific suPAR hextiles. Data from the emergency departments at Copenhagen University Hospital Hvidovre and North Zealand Hospital (*n* = 9591) ([19]; Rasmussen et al.: suPAR in Acute Care: Associated with Disease Severity, Readmission, and Mortality, in review). SuPAR = soluble urokinase plasminogen activator receptor

**Fig. 2:**
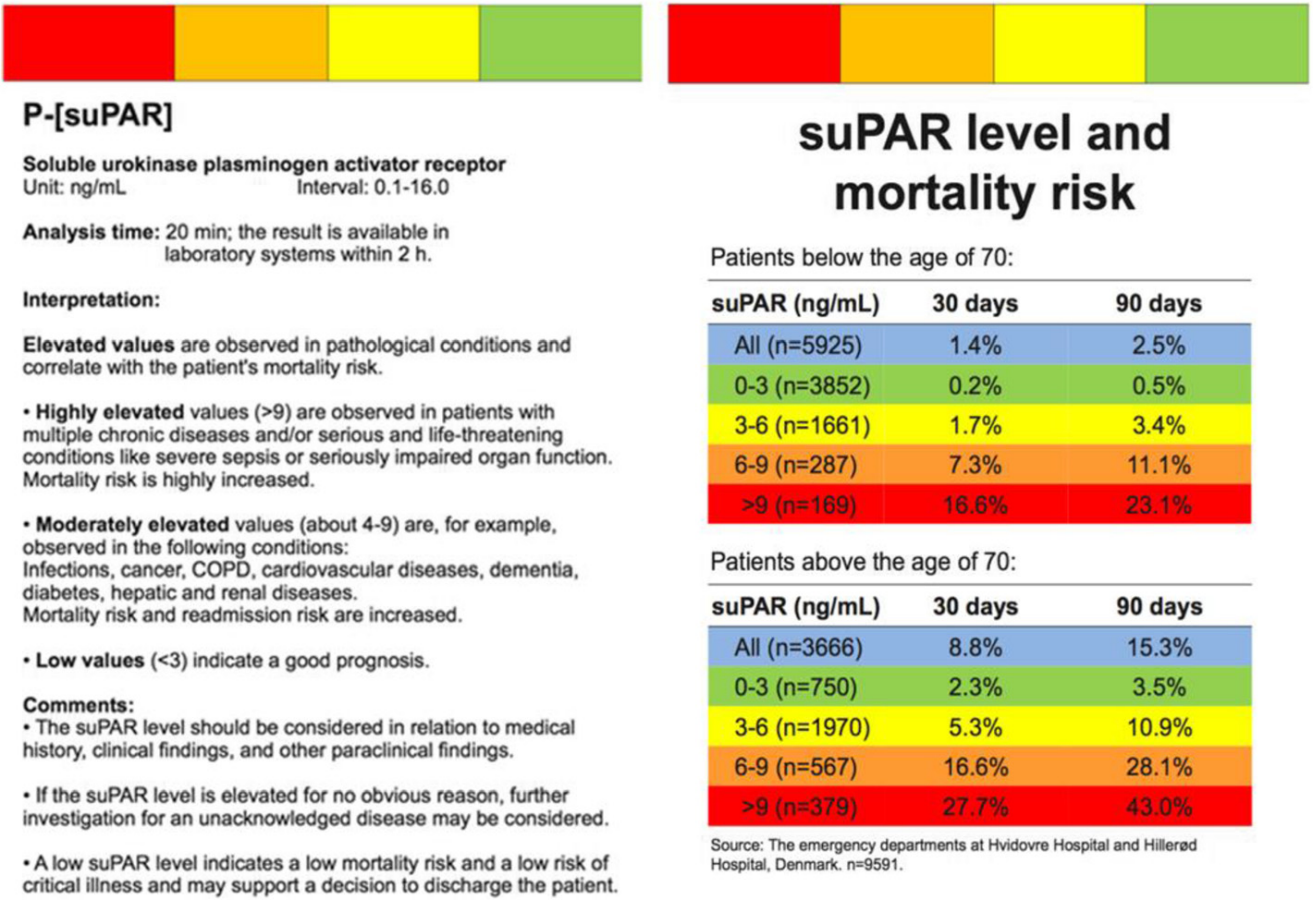
Pocket card given to all physicians in the ED illustrating suPAR level interpretation and mortality risk stratified by suPAR intervals. ED = emergency department, suPAR = soluble urokinase plasminogen activator receptor, COPD = chronic obstructive pulmonary disease

Prior to the study, all physicians working in the emergency department have been informed in writing about the prognostic abilities of suPAR in unselected patients, and in regard to specific diagnoses in the form of a review of published literature, as well as pocket cards providing unadjusted mortality rates from 10.000 patients from similar EDs (see Figs. 1 and 2) ([19]; Rasmussen et al.: suPAR in Acute Care: Associated with Disease Severity, Readmission, and Mortality, in review). Furthermore, the physicians working at each specialty in the ED have attended presentations where the prognostic significance of suPAR levels and associations with morbidity and mortality were elaborated.

To assess the quality of our data, and whether the physicians received and considered the suPAR level in the initial evaluation of patients, a questionnaire is sent to 200 randomly selected physicians at the participating hospitals (Table 3).

**Table 3:** Questions included in the electronical questionnaire

Did you see the suPAR level of your patient?	
Did you feel informed in the prognostic ability of suPAR?	
How often did you include suPAR in your combined assessment of your patient?	
How often did the suPAR level influence your clinical decision?	
How often were you surprised of a high suPAR level?	
How often were you surprised of a low suPAR level?	

*SuPAR* soluble urokinase plasminogen activator receptor

#### Data collection

Results of blood sample analyses including suPAR level will be obtained from the LABKA II database. Using the unique Danish central person registration number (CPR-number), data will be transferred to “Statistics Denmark” [22] and merged with data from central registries. Demographic data and mortality will be obtained from the Central Civil Registry where all residents in Denmark are registered. Data on admissions, discharges, and diagnoses will be obtained from the National Patient Registry (NPR). NPR contains information coded according to the International Statistical Classification of Disease, 10th revision (ICD-10) on primary diagnosis of discharge (A-diagnosis) and comorbidity (B-diagnoses). In the data analysis, the suPAR level from the index admission will be linked with the data above to examine the primary and secondary outcomes.

#### Power calculation

In a previous unpublished study of patients acutely admitted to the ED at Copenhagen University Hospital North Zealand, Denmark, 12-month mortality was 12.7 % and the frequency of readmissions was 16 % within 5 months.

The main hypothesis is to assess if all-cause mortality at 10 months after admission is lower when the biomarker is measured on acutely admitted patients. Using a 5 % level of significance and a power of 80 %, we will need a sample of 7340 patients in each randomization group to detect an absolute risk reduction in mortality at least 10 months after admission of 1.5 %.

The emergency departments at Copenhagen University Hospital Herlev and Copenhagen University Hospital Bispebjerg have a catchment area of 447.000 and 454.000 inhabitants, respectively. Based on the figures from the EDs of the hospitals included, approximately 170 patients are admitted on a daily basis. During the intervention cycles, approximately 10.710 patients will be admitted and available for suPAR measurements while 10.710 patients will be admitted in the control cycles. Thus, we anticipate to recruit 47 % more patients than what is required with individual randomization, and this increase is deemed sufficient to account for intra-cluster dependency.

#### Statistical analysis

Patients admitted in each intervention or control cycle will be followed as a single cohort and data will be analyzed as randomized. The two groups will be assessed for comparability of the following variables: age, sex, and Charlson score. Differences in mean age of more than 5 years and/or an absolute Charlson Comorbidity Index score of 2 or more will be adjusted for in the final analysis.

Patient data will be analyzed according to what arm of the trial the patient is admitted to during index admission according to the randomization scheme (Table 1) corresponding to the intention-to-treat principle.

A weighted Cox model will be used to compare mortality at 10 months after inclusion of the last patient. Patients are censored if their first readmission is in the opposite group of their index admission. As this censoring is likely to be dependent censoring (a readmission is rarely a positive prognostic signal), we will employ Inverse Probability of Censoring Weighting (IPCW) where patients readmitted to their own treatment group will be up-weighted to compensate. We will employ stabilized weights such that the reweighted sample has the same implied sample size throughout follow-up. Due to the design, time since index admission is the only covariate that needs to be included in the weights. Reweighing will be done for every two weeks of follow-up. We will not censor nor reweight for 2nd or later readmissions, since the weights would become highly unstable and it is not likely that the presence or absence of an initial suPAR measurement will be important for clinical decisions at this stage.

Furthermore, a traditional intention-to-treat analysis will be performed. Notable difference between the results of the two analysis strategies will be considered critically.

Kaplan-Meier plots will be used to illustrate survival. Unpaired T-test will be used to compare length of stay. *P* < 0.05 will be considered significant.

Subgroup analysis of the following groups will be performed: patients aged 65 year and above, and patients discharged with diagnoses of surgical conditions, cancer, infections, and cardiovascular disease.

## Discussion

Rapid and safe risk stratification is necessary and an important task in emergency medicine. Identifying patients at high and low risk shortly after admission can guide clinical decision-making towards the patients in need, regarding treatment, observation and allocation of resources. Several studies have suggested biomarkers as a supplement to enhance risk stratification; however they have only been studied retrospectively [3–7, 9, 10], why an interventional study is both warranted and required, in order to quantify the effects of implementing a prognostic biomarker in emergency medicine. The current study is to our knowledge the first of its kind, and focuses on whether the availability of a prognostic biomarker influences the treatment strategy and overall prognosis of patients admitted to the ED.

SuPAR has been evaluated as a potential biomarker in the general population by 5 large studies with more than 4500 randomly selected healthy participants which showed that elevated suPAR is associated with an increased risk of cardiovascular morbidity and mortality [13, 14, 23–25]. The TRIAGE I study along with others found suPAR independently associated with short-term mortality [12, 18, 19], and when analyzing prospective data of the TRIAGE I study, the supplementary prognostic information of suPAR was evident (Rasmussen et al.: suPAR in Acute Care: Associated with Disease Severity, Readmission, and Mortality, in review). Receiver operational characteristic (ROC) analysis in relation to 30-day mortality showed an AUC of 0.85 (95 % CI 0.82-0.87) when triaging with suPAR and 0.62 (0.58-0.66) when triaging with the usual triage algorithm based on vital signs and symptoms [19, 26]. Several biomarkers have shown to carry prognostic value, for example C-reactive protein, YKL-40, Pentraxin-3, and Copeptin [26]. SuPAR was chosen in our current interventional study based on its superiority in the TRIAGE I study, which indicated that suPAR might be a very good candidate for risk stratification in the emergency setting [19].

SuPAR as a biomarker is reflective of low-grade inflammation, a key component of disease development in e.g. cancer, cardiovascular, renal, and infectious disease and it is also strongly associated with the Charlson Comorbidity Index [11–17, 27–34]. Plasma levels of suPAR are associated with factors such as smoking, alcohol consumption, and a passive lifestyle [14]. Previous studies have shown that the urokinase system is deeply integrated in the pathogenesis of atherosclerotic remodeling and plays a role in fibrinolysis, angiogenesis and immunologic function [29, 35–37]. The suPAR level reflects immune activation and the inflammatory state of the individual. The protein uPAR is primarily expressed on immunological cells like monocytes, leukocytes, endothelial cells and is thought to reflect subclinical organ damage and endothelial dysfunction [13, 25, 38]. SuPAR meets many basic biochemical criteria of an ideal biomarkers because it is stable in plasma and is not significantly affected by the circadian cycle [39].

A biomarker reflecting the level of urgency or comorbidity burden could potentially be very useful, but the value of a biomarker with a strong negative predictive value must not be underestimated [12]. The availability of a biomarker reflecting healthiness or non-urgency (e.g. low plasma suPAR level) is particularly interesting in the setting of emergency departments where crowding is a serious concern. High bed occupancy rates are associated with an increased mortality rate, delays in initiation of time-critical care and diagnosis, increased costs and an overall poor quality of care and concerns of patient safety [40–42]. Furthermore, hospitalization is associated with a number of adverse outcomes such as falls, medication errors, infections, and delirium [43, 44]. Early discharge is associated with decreased mortality and increased patient outcome, illustrated by an American and a British study that found 26 % respectively one fifth of all hospitalizations were potentially avoidable [45–47], why a more efficient selection of patients without need of admission is desirable.

### Strengths and limitations

The strength of this trial is the large size of the cohort, multicenter nature and inclusion of a heterogeneous cohort under a wide variety of medical and surgical settings. Due to the unselective cohort, the trial brings generalizability whereby results will be applicable to nearly all patients admitted to EDs. A learning curve for physicians must be expected when implementing a prognostic biomarker in terms of suPAR level interpretation and intervention, why physicians might refrain from discharging patients on the basis of low suPAR levels because they do not fully trust the prognostic abilities of suPAR.

In the planned data analysis of the primary outcome, readmitted patients remain in the group to which they are primarily allocated (control vs. suPAR intervention). This increases the risk of a type 2 error as patients primarily included in the control group might have one or more following admissions where they have suPAR measured and vice versa. A possibility would be to exclude all patients that were admitted more than once during the inclusion. If this method was chosen, we would risk excluding the sickest patients, where suPAR might have the greatest value.

## Conclusion and clinical implications

The TRIAGE III trial has the potential to investigate the concept of whether the availability of prognostic information can change the patient’s prognosis. This concept is central in triage and several other clinical situations, and might therefore have a central impact on health care organization and decision-making. If our hypothesis is confirmed, considerations should be given towards standardizing prognostic biomarkers as routine blood work in relation to early risk stratification in the ED.

## Abbreviations

COPD, chronic obstructive pulmonary disease; ED, emergency department; suPAR, soluble urokinase plasminogen activator receptor
